# Quantitative chromatin proteomics reveals a dynamic histone post-translational modification landscape that defines asexual and sexual *Plasmodium falciparum* parasites

**DOI:** 10.1038/s41598-017-00687-7

**Published:** 2017-04-04

**Authors:** Nanika Coetzee, Simone Sidoli, Riëtte van Biljon, Heather Painter, Manuel Llinás, Benjamin A. Garcia, Lyn-Marie Birkholtz

**Affiliations:** 1 Department of Biochemistry, Institute for Sustainable Malaria Control, University of Pretoria, Private bag x20, Hatfield, Pretoria South Africa; 2 Epigenetics Program, Department of Biochemistry and Biophysics, Perelman School of Medicine, University of Pennsylvania, Philadelphia, Pennsylvania USA; 3 Department of Biochemistry & Molecular Biology and Centre for Malaria Research, Pennsylvania State University, University Park, Pennsylvania, USA

## Abstract

Gene expression in Plasmodia integrates post-transcriptional regulation with epigenetic marking of active genomic regions through histone post-translational modifications (PTMs). To generate insights into the importance of histone PTMs to the entire asexual and sexual developmental cycles of the parasite, we used complementary and comparative quantitative chromatin proteomics to identify and functionally characterise histone PTMs in 8 distinct life cycle stages of *P. falciparum* parasites. ~500 individual histone PTMs were identified of which 106 could be stringently validated. 46 individual histone PTMs and 30 co-existing PTMs were fully quantified with high confidence. Importantly, 15 of these histone PTMs are novel for Plasmodia (e.g. H3K122ac, H3K27me3, H3K56me3). The comparative nature of the data revealed a highly dynamic histone PTM landscape during life cycle development, with a set of histone PTMs (H3K4ac, H3K9me1 and H3K36me2) displaying a unique and conserved abundance profile exclusively during gametocytogenesis (*P* < 0.001). Euchromatic histone PTMs are abundant during schizogony and late gametocytes; heterochromatic PTMs mark early gametocytes. Collectively, this data provides the most accurate, complete and comparative chromatin proteomic analyses of the entire life cycle development of malaria parasites. A substantial association between histone PTMs and stage-specific transition provides insights into the intricacies characterising Plasmodial developmental biology.

## Introduction

Epigenetic heritage describes the ability of cells to transmit chromatin structural modifications during cell division and thereby maintaining differentiation status in eukaryotes. Histone proteins and their variants mediate this process and are modified through a myriad of post-translational modifications (PTMs) such as acetylation, methylation, phosphorylation, SUMOylation and ubiquitination. Histone modifications impact DNA regulatory processes during normal cell cycle progression including transcriptional activation, DNA replication, DNA damage repair and chromosomal condensation^1^. The levels of histone PTMs are regulated by reader, writer and eraser proteins including families of histone methyltransferases (HMTs), demethylases, histone acetyltransferases (HATs) and deacetylases (HDACs). Histone acetylation is associated with gene activation in a transcriptionally permissive state, whereas methylation results in repression or activation, in a position-dependent manner^2^. Although histones and their modifying proteins are conserved between different organisms, they contain alternative sites for modification within early-branched Eukarya, mediated by divergence of the modifying proteins^3, 4^. This reveals evolutionary unique functions for these histone PTMs in gene expression regulation and other chromatin-mediated processes involved during development^5–9^.

Within the Apicomplexans, malaria parasites still caused mortality in 438,000 individuals in 2015^10^. These parasites have an extremely complex life cycle consisting of two distinct developmental phases including asexual proliferation and sexual progression. The characteristic pathogenesis associated with the disease is due to a massive increase in parasite numbers (e.g. *Plasmodium falciparum*) during their asexual amplification in human erythrocytes. However, a minor portion (<10%) of these parasites are able to undergo sexual differentiation with every asexual cycle^11^, forming male and female gametocytes that ensure continued parasite transmission to female Anopheline mosquitoes. Five distinct morphological forms of development are distinguishable during sexual maturation in humans. Gametocytogenesis is a non-replicative cellular differentiation process taking ~10–14 days^12^ and resulting in a nearly quiescent phenotype, awaiting activation in the mosquito midgut^13^. Gametocyte maturation is characterised by transcriptional differentiation^14^ and changes in energy and lipid metabolism^15^. Commitment to gametocytogenesis has recently been directly linked to epigenetic control, regulated by the AP2-G transcription factor^16, 17^. Disruptors of heterochromatin silencing have also been associated with gametocyte overproduction, leading to the identification of molecular players involved in epigenetics, including heterochromatin protein 1^18^ and histone deacetylase 2^19^.

Developmental regulation in Plasmodia involves stringent transcriptional control through the expression of the majority of genes only at particular points during development^14, 20^. The parasite, therefore, relies on a complex program of regulation of gene expression^20–22^, integrating post-transcriptional regulation with specific transcription factors, histone positioning, expression of histone variants and histone PTMs^5, 23^. During the asexual, intra-erythrocytic developmental cycle (IDC), a large proportion of the Plasmodial genome is acetylated and found in a predominantly euchromatic, transcriptionally permissive chromatin state^24–27^. The meticulous characterisation of the full cascade of histone PTMs (and the associated effector proteins) throughout the parasite’s life cycle is a crucial first step towards identifying epigenomic ‘check-points’ in the cell cycle and sexual development in *P. falciparum* parasites^28^. For instance, HDACs and HMTs as effector proteins play critical roles in the parasite’s maturation throughout the IDC^29–36^. Histone PTMs are associated with cell type-specific proteins in other organisms and have a high heritability during cell division^37^. This leads to the establishment of a global chromatin environment that contributes to the regulation of transcriptional expression^1, 38^. The type and position of a specific PTM, as well as the combination of PTMs, play a crucial role in determining its biological relevance in DNA-dependent biological processes^37^.

The four core histones H2A, H2B, H3, and H4, as well as four *P. falciparum* variant histones H2A.Z, H2Bv (or H2B.Z), H3.3 and H3Cen (H3 centromeric), have previously been identified, but linker histone H1 has not been identified in *P. falciparum* parasites^39, 40^. Histone PTMs (including acetylation, methylation, etc.) have been qualitatively identified for asexual stage parasites^1, 21, 31, 39–49^, with a single recent quantitative analyses during asexual replication using a mass spectrometry (MS)-based strategy that combines spectral counting and validation by targeted acquisition^50^. Only twelve modifications have been linked with the dynamic transcriptional pattern during asexual development through genome-wide localisation^21, 51^. However, information of the involvement of individual histone PTMs in progression through specific compartments throughout the entire asexual and sexual life cycle is still incomplete. Notably, information on histone PTMs in the sexual gametocyte forms of the parasite is limited to the identification of only two histone PTMs in inhibitor studies^52^.

Given the relevance of histone PTMs to malaria parasite development and survival, profiling of the complete histone PTM landscape across the entire *P. falciparum* life cycle is, therefore, essential. Here, we report the first extensive and fully quantitative analysis of the complete histone PTM landscape across eight distinct life cycle stages of *P. falciparum* parasites, spanning the entire asexual and sexual development stages. To do so, we applied quantitative, high-resolution MS combined with nano liquid chromatography (nanoLC) and computational analysis using canonical database searching and our in-house developed software for accurate LC peak area extraction^53^. We demonstrate that histone PTM signatures differentiate the asexual (ring, trophozoite and schizont) from sexual (stage I–V gametocytes) developmental stages. Novel histone PTMs were identified, some of which are enriched in specific life cycle forms of the parasite. A proportion of the histone PTMs (e.g. H3K4ac, H3K9me3, H3K36me2, H3K122ac, H4ac and H4K20me3) show a marked dynamic nature within both asexual and sexual development. This paper thus provides an enhanced understanding of the unique developmental cascade present in *P. falciparum* parasites, especially during the IDC, host-adaptation and sexual differentiation of these parasites.

## Results

### Histone abundance profile during development

**Figure 1:**
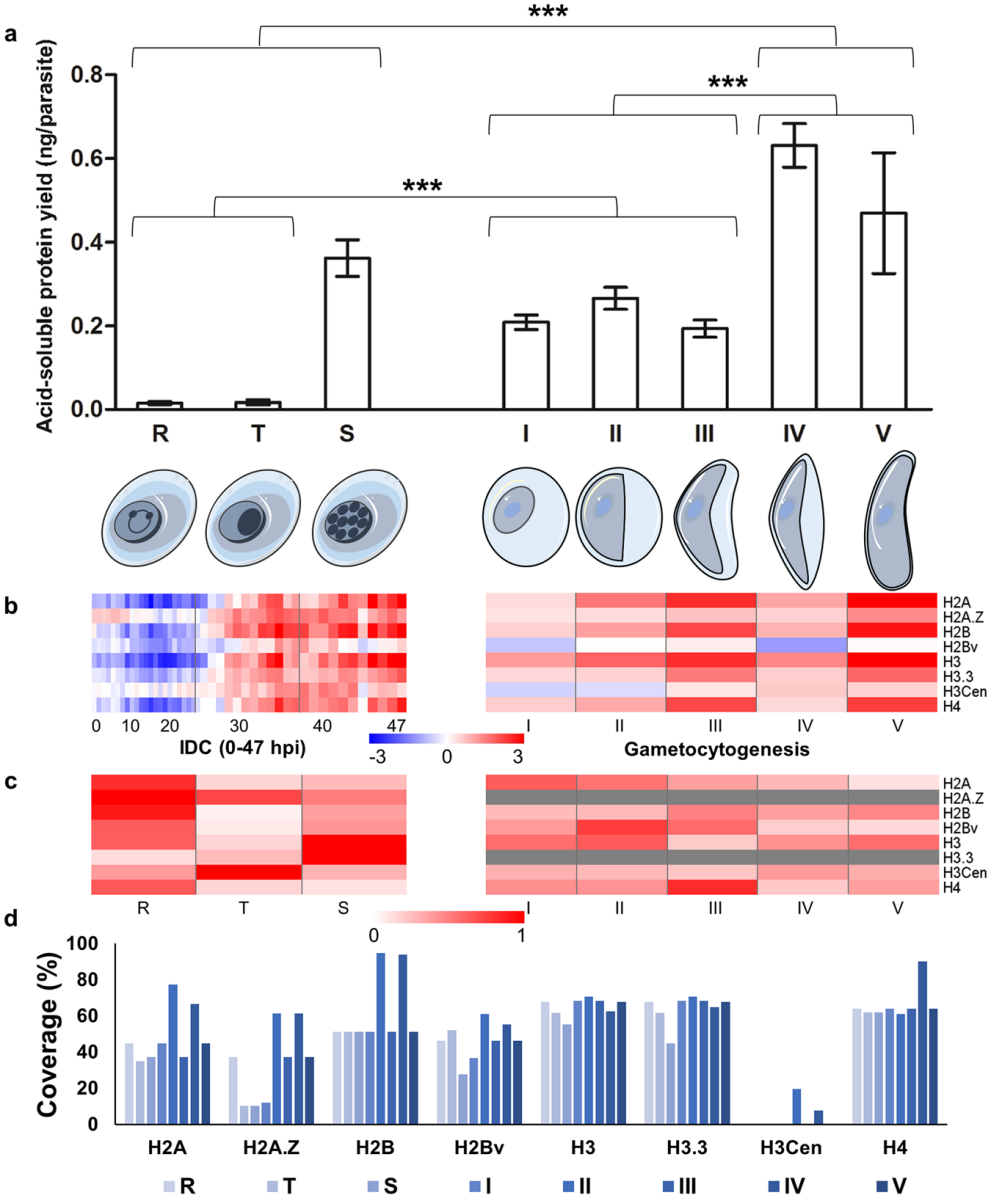
*P. falciparum* histone prevalence during development. Eight *P. falciparum* life cycle stages, including rings, trophozoites, schizonts and stage I, II, III, IV and V gametocytes were isolated and the histones were acid-extracted. (**a**) The yield (ng/parasite) of histone-enriched, acid-soluble protein fractions per isolated parasite for each developmental stage was determined (two-tailed equal variance t-test, ***P < 0.001, n = 3 ± SEM). Histones refer to the isolated acid-soluble protein fraction containing the histone proteins and not to pure isolated histones. (**b**) Expression of the eight *P. falciparum* histones over asexual and sexual life cycle development. The expression values (log_2_, Cy5/Cy3) of the core and variant histone transcripts are shown over the 48 h IDC (hpi) and throughout the duration of gametocytogenesis (days). (**c**) Protein abundance of the eight *P. falciparum* histones over asexual and sexual life cycle development. The relative abundance (normalised %) of the core and variant histone proteins are shown over the IDC and gametocytogenesis. The relative abundance data for H2A.Z and H3.3 was not available (indicated by grey bar). (**d**) Histone sequence coverage (%) for the eight *P. falciparum* histones are shown for each life cycle stage. H3Cen was only identified in stage II and IV gametocytes.

To identify histone PTMs throughout asexual and sexual development of malaria parasites, we isolated histones from chromatin extracts of the parasites at eight life cycle stages: ring, trophozoite and schizont in asexual development (>90% synchronised) and stage I through V of gametocytogenesis (>60% stage I; >50% stage II; >80% stage III; >90% stage IV; and >85% stage V; see Supplementary Figure S1). This allowed for the identification of all core histones typically present in *P. falciparum* parasites, including their variants (Fig. 1). The yields of the histone-enriched, acid-soluble nuclear protein fractions differed between the various life cycle stages (Fig. 1a) with the lowest yield observed during early asexual development (rings at 0.015 ± 0.006 ng total histones/parasite; trophozoites at 0.017 ± 0.011 ng/parasite; *P* > 0.8, n = 3) and a significant increase observed in schizonts (0.36 ± 0.08 ng/parasite; *P* < 0.001, n = 3). This showed similar trends compared to histone gene expression, transcriptional activity and DNA synthesis during schizogony^6, 28, 40^. By comparison, the total yields of the histone-enriched fractions for gametocytes were significantly higher (*P* < 0.001, n = 3) than that of early asexual stage parasites (stage I at 0.208 ± 0.030 ng/parasite, stage II at 0.27 ± 0.05 ng/parasite, stage III at 0.19 ± 0.04 ng/parasite) and the yield of late gametocyte development (stage IV at 0.63 ± 0.09 ng/parasite and stage V at 0.47 ± 0.25 ng/parasite; *P* < 0.001, n = 3) was also significantly higher than during early gametocyte development, although the yields for these two stages (IV and V) were not statistically different from each other (*P* > 0.05).

The yields of the histone-enriched fraction showed similar trends compared to increased gene expression levels of the histones observed during schizogony and gametocyte development (Fig. 1b), clearly peaking in the mature developmental stages of *P. falciparum* parasites. The variant histones were shown to have an overall lower expression than the core histones, peaking in mature asexual development. Interrogation of the relative abundance levels of the histones showed that these proteins are present throughout asexual and sexual development, with some histones peaking in different developmental stages (Fig. 1c). These increased expression levels of histones in mature gametocytes occur against a background of general decreased RNA and protein synthesis^14, 54, 55^, with no translational repression seen for histones^56^.

### Complete quantitative histone PTM landscape of asexual and sexual *P. falciparum* parasites

We performed a quantitative assessment of histone samples using high-resolution nanoLC-MS/MS; data processing/peak area extraction was performed by using EpiProfile^53^, and spectra identification was performed by using Proteome Discoverer (Thermo). This enabled confident identification and quantification of histone PTMs, as well as horizontal comparison of the histone PTM landscape during *P. falciparum* parasite development. For the quantitative chromatin proteomics study, a complete validated dataset of 125 individual peptides were confidently identified (<20% coefficient of variance between biological replicates), of which 17% constituted the unmodified proportion of the peptides (see Supplementary File S1). Reproducibly high sequence coverage was obtained in all three biological replicates, particularly for the core histones at 61 ± 15% (Fig. 1d). In total, the variant histones were associated with lower sequence coverage compared to their core partners (H2A.Z vs. H2A, *P* = 0.84; H2Bv vs. H2B, *P* = 0.69). As expected, due to the centromere-restricted localisation of H3Cen^57^, this protein was present in low abundance with significantly lower sequence coverage (*P* < 0.01) than any of the other histones.

The high mass accuracy (0.8 ppm), as well as manual curation, enabled confident discrimination between modifications such as acetylation (42.011 Da) and trimethylation (42.047 Da). Our in-house software EpiProfile also includes features like intelligent retention time evaluation, i.e. uses previous knowledge about the order of elution from LC of differently modified peptides, and uses MS/MS fragment profiles to discriminate the abundance of isobaric and co-eluting histone peptides (see Supplementary Figure S2)^53^. By using this approach, we could evaluate all MS peaks and quantify the presence of specific histone PTMs.

**Figure 2:**
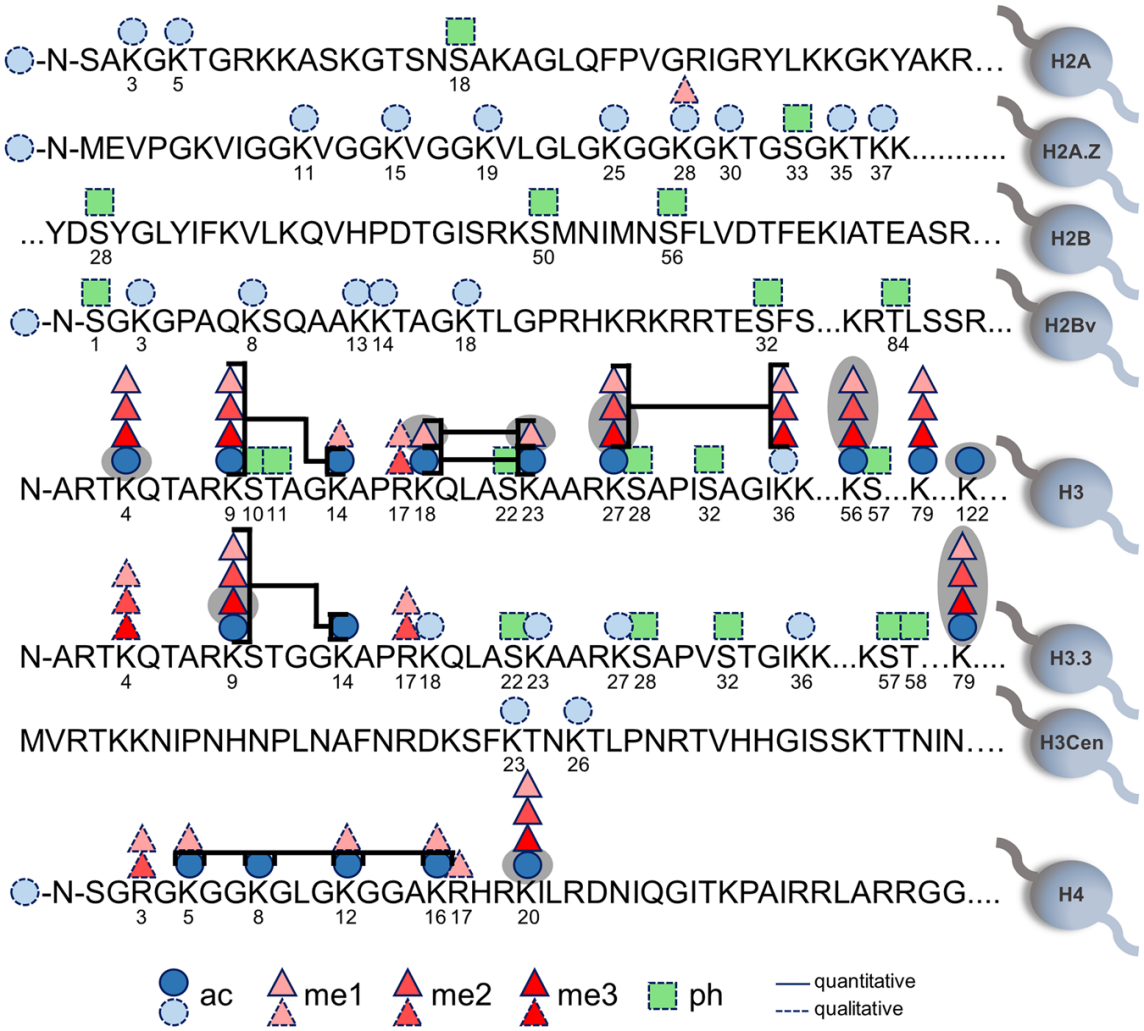
*P. falciparum* histone PTM landscape. A wide array of histone PTMs have been identified in *P. falciparum*, including 15 novel PTMs that were identified for the first time in this study (shaded grey). Five principles types of histone PTMs have previously been reported, including acetylation, methylation (mono-, di- and tri-), phosphorylation, ubiquitination and SUMOylation. In this study, 106 PTMs were identified (dashed line), but only 46 of these were quantifiable in all stages over life cycle development (solid line). Co-existing histone PTMs on individual peptides were also identified (brackets).

Initial analysis revealed a total of 493 histone PTMs over all the life cycle stages (Supplementary File S2), after which the spectra were manually validated and the resulting semi-quantitative histone PTM landscape of *P. falciparum* parasites contained 106 individual validated histone PTMs (Fig. 2 and Table 1). This included the majority of the histone PTMs that have previously been identified in *P. falciparum* but certain modifications that have been previously reported (H2AK5sumo, H2AK20sumo, H2AK36sumo, H2AS104ph, H2AS120ph, H2AK123sumo, H2AS126ph, H2BK112ub and H3K37ub) were not detected in this study^39, 46, 49^. Moreover, only qualitative identification of 19 individual phosphorylation sites was obtained. Enrichment for SUMOylation and ubiquitination was not performed to maintain the integrity of the isolated histone samples; the dataset therefore does not contain any information on these modifications.

**Table 1:** Histone PTMs identified in *P. falciparum* parasites.

Histone	Acetylation	Methylation			Phosphorylation
		Mono-	Di-	Tri-	
H2A	N-term, K3, K5				S18
H2A.Z	N-term, K11, K15, K19, K25, K28, K30, K35, K37	K28			S33
H2B					S28, S50, S56
H2Bv	N-term, K3, K8, K13, K14, K18				S1, S32, T84
H3	**K4**, **K9**, **K14**, **K18**, **K23**, **K27**, K36, **K56**, **K79**, **K122**	**K4**, **K9**, K14, R17, **K18**, **K23**, **K27**, **K36**, **K56**, **K79**	**K4**, **K9**, R17, **K27**, **K36**, **K56**, **K79**	**K4**, **K9**, **K27**, **K36**, **K56**, **K79**	S10, T11, S22, S28, S32, S57
H3.3	**K9**, **K14**, K18, K23, K27, K36, **K79**	K4, **K9**, R17, **K79**	K4, **K9**, R17, **K79**	K4, **K9**, **K79**	S22, S28, S32, S57, T58
H3Cen	K23, K26				
H4	N-term, **K5**, **K8**, **K12**, **K16**, **K20**	R3, K5, K12, K16, R17, **K20**	R3, **K20**	**K20**	

Regular font indicates qualitative PTMs identified in this study (also previously identified).

Bold indicates quantitative PTMs identified in this study (also previously identified).

Bold & underlined indicates quantitative PTMs identified in this study (novel PTMs).

To allow accurate and comparative data across the life cycle stages, stringent quantification and validation was performed on the 106 modifications identified. This resulted in 55 modifications that could be accurately quantified using EpiProfile (Fig. 2, Table 1) and from these, 46 histone PTMs were validated and therefore represents the complete and comparative quantitative histone PTM landscape for *P. falciparum* parasites across its entire life cycle in human blood (Supplementary File S3). These individual modifications occurred mostly on histone H3 and its variant H3.3, followed by H4 with the third most prevalent number of modifications. These histones are known to be highly susceptible to modification. Comparatively, very few modifications were observed on H2A and H2B (and their variants) as well as H3Cen, and none of these modifications could be quantified, due to their low abundance status in the total population.

The histone PTM landscape contained quantitative identification of 26 histone PTMs that have previously been only qualitatively described by MS based approaches (Fig. 2, Table 1)^21, 31, 39, 40, 42, 43, 45, 46, 48, 49^. However, in total, 15 histone PTMs were additionally quantitatively identified for the first time in *P. falciparum* (Fig. 2, Table 1). These novel marks included 6 modifications that are known to be involved in transcriptional activation (H3K4ac, H3K122ac) and repression (H3K27me2, H3K27me3, H3K56me3, H3.3K9me3) based on their functions in other model organisms^58–65^. In addition to the set of unique, single histone PTMs, we also identified 39 individual peptides that contained conclusive data for the co-existence of histone PTMs on neighbouring residues (Fig. 2, see Supplementary File S4). This included 30 validated combinations of all modifications on H3K9 (ac, me1, me2 & me3) with H3K14ac, with the same combinations seen for histone H3.3. Additional combinations include H3K18ac/me1 & H3K23ac/me1, H3K27me1-3 & H3K36me1-3 and H4ac at K5, K8, K12 and K16.

### Dynamic histone PTM profiles between asexual and sexual development

**Figure 3:**
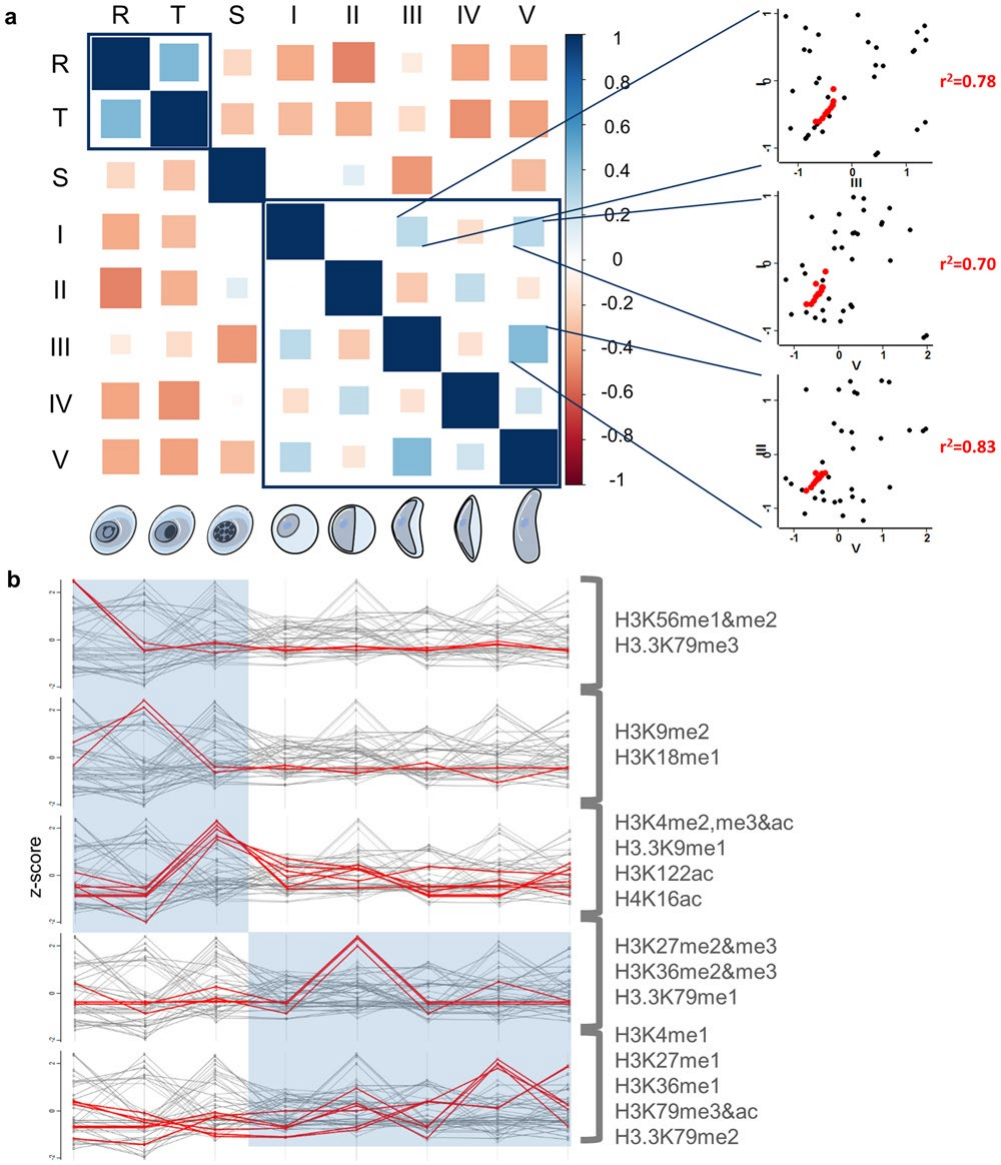
Dynamic patterns of histone PTMs between asexual and sexual development. (**a**) Correlation of the overall histone PTM landscape between the eight *P. falciparum* life cycle stages shows an early asexual cluster and a gametocyte cluster of correlating PTM landscapes (Pearson correlation). Correlation of a subset of 13 conserved residues (H3K4ac, H3K9me1, H3.3K9me2, H3K18me1, H3K23me1, H3K27me2&me3, H3K36me2&me3, H3K56me1&me2, H3K79me3 and H3.3K79me3) between stage I & III (r^2^ = 0.78), I & V (r^2^ = 0.70) and III & V (r^2^ = 0.83) gametocytes are shown in red. (**b**) Stage-specific trends based on z-score distribution are shown for rings (H3K56me1&me2; H3.3K79me3), trophozoites (H3K9me2; H3K18me1), schizonts (H3K4me2, me3 & ac; H3.3K9me1; H3K122ac; H4K16ac) early gametocytes (H3K27me2 & me3; H3K36me2 & me3; H3.3K79me1) and late gametocytes (H3K4me1; H3K27me1; H3K36me1; H3K79ac & me3; H3.3K79me2).

The most significant observation in this study is the dynamics and plasticity of histone PTMs as the parasite differentiates during asexual and sexual development. The quantitative dataset allowed head-to-head comparison of the changes in the histone PTMs across all stages (ring, trophozoite and schizont) of the *P. falciparum* parasite’s asexual developmental cycle, as well as all five distinct morphological stages (stage I–V) of sexual gametocytogenesis. A close positive correlation (r^2^ = 0.44, Fig. 3a) was observed between early asexual stages (ring and trophozoites). However, the transcriptionally active and differentiating schizont stage was diverged from early asexual development, as expected. Gametocyte histone PTMs is in totality divergent from that found in asexual parasites, with certain gametocyte stages showing closer total correlation (e.g. stage III & V: r^2^ = 0.43, Fig. 3a). Within these gametocyte stages, a subset of 13 conserved modifications (H3K4ac, H3K9me1, H3.3K9me2, H3K18me1, H3K23me1, H3K27me2&me3, H3K36me2&me3, H3K56me1&me2, H3K79me3 and H3.3K79me3) was highly correlated between stage I & III (r^2^ = 0.78), I & V (r^2^ = 0.70) and III & V (r^2^ = 0.83) gametocytes (Fig. 3a). Such a pattern of highly correlated and conserved modifications within the subset of 13 modifications was only observed in certain sexual stages but not in asexual parasites or stage II/IV gametocytes (see Supplementary Figure S3).

Further delineation of this stage divergence indicated subsets of modifications that have increased abundance in specific life cycle compartments (Fig. 3b). Of these, H3K56me1&me2 and H3.3K79me3 showed peak abundance in transcriptionally inactive ring stage parasites, while transcriptional activation is associated with the presence of H3K9me2 and H3K18me1 in trophozoites. The number of stage-specific modifications increases 3-fold in schizont development, including H3K4me2, me3&ac, H3.3K9me1, H3K122ac and H4K16ac. By contrast, gametocytes are associated with a set of modifications that are quite distinct from those observed in asexual parasites, indicating diverging functionality. These PTMs include H3K27me2&me3, H3K36me2&me3 and H3.3K79me1 that peak in early stage gametocytes whilst H3K4me1, H3K27me1, H3K36me1, H3K79me3&ac and H3.3K79me2 abundance are exclusively increased in late stage gametocytes.

**Figure 4:**
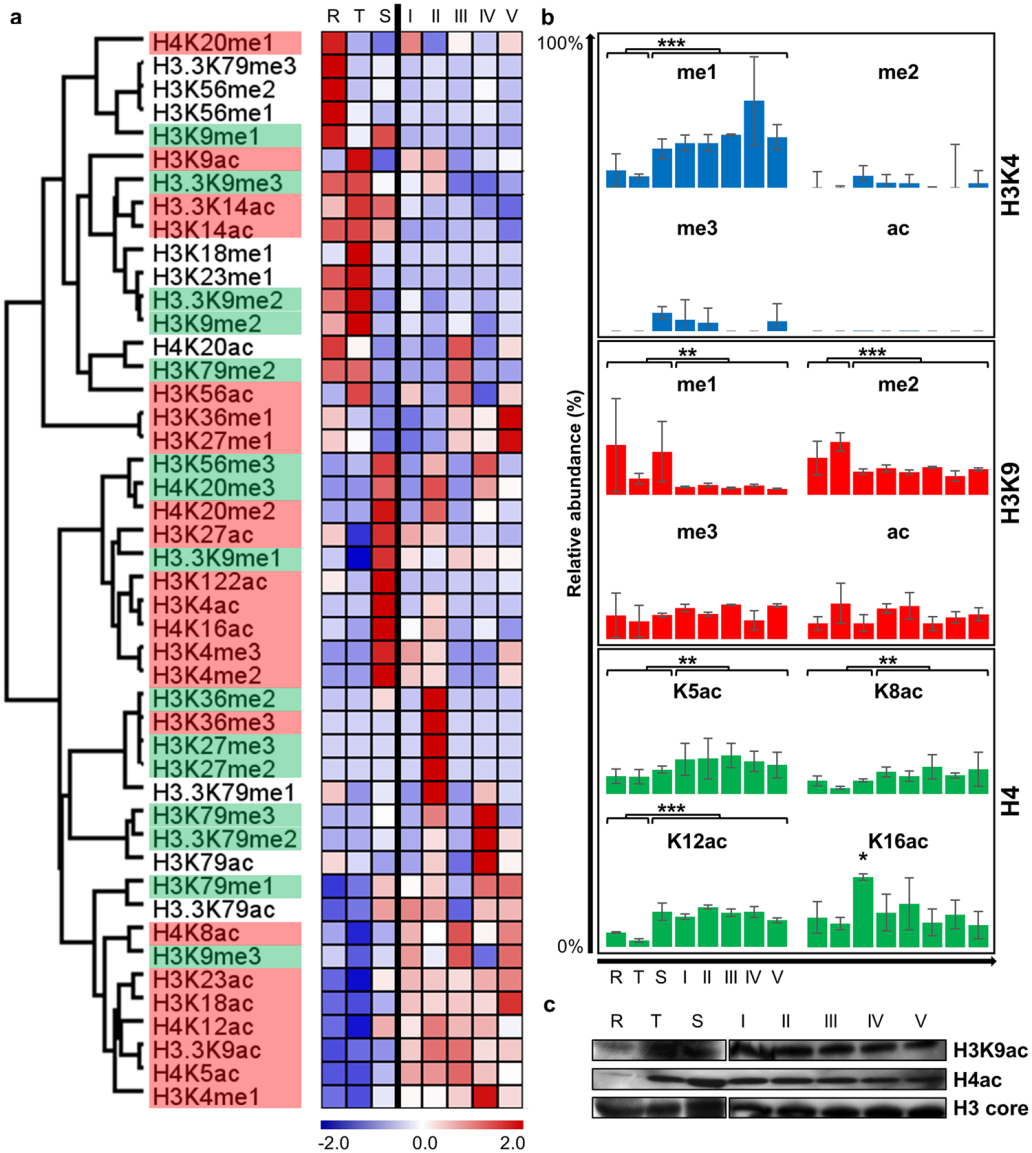
Patterns of gene activation and silencing during life cycle development. (**a**) The histone PTM relative abundance (z-score) over the life cycle is shown (ranging from R, T, S, I, II, III, IV and V from left to right). Z-scores were clustered hierarchically, uncentered (similar results obtained with K-means) with Pearson Correlation on average linkages, and subsequently phase ordered. Euchromatin (red) or heterochromatin (green) status for certain histone PTMs are shown where the information has previously been confirmed in *P. falciparum* or based on involvement in other model organisms^58, 70–76^. (**b**) The me1, me2, me3 and ac relative abundance levels (%) over life cycle development is shown for histone H3K4, H3K9 and H4 (***P < 0.001; **P < 0.01; *P < 0.05; n = 3 ± SEM). (**c**) Histone H3K9ac, H4ac (K5 + K8 + K12 + K16) and histone H3 (core) levels were independently investigated using immunoblotting over the eight harvested life cycle stages, validating overall lowered levels during rings (except for H3 core) and higher levels from trophozoites and schizonts through gametocytogenesis. Blots were cropped for this image (full-length blots are presented in Supplementary Figure S4). All samples were derived from the same experiments and gels/blots were processed in parallel.

We subsequently interrogated the stage-specific distribution of the individual histone PTMs to the life cycle compartments. Global analysis of the dynamic nature of the histone PTM landscape indicated a phase-like distribution via hierarchical clustering (Fig. 4a), reminiscent of differential transcriptional expression during asexual development^20^. Histone PTMs are therefore not equally distributed across the entire asexual and sexual development, but stage-specific histone PTM abundance profiles were observed. However, the stage-specific distribution of the known histone PTMs for euchromatin (activation modifications H3K4me1/2/3, H3K9ac, H3K14ac, H3K27ac & H4ac) and heterochromatin (repressive modifications H3K9me3, H3K79me3, H4K20me3, H3K27me3)^29, 30, 32, 34, 36, 40, 42, 45, 57, 59–63, 65^, showed that, even though the relative abundance of these marks may vary, all of these modifications are present in all the life cycle stages, confirming their importance and involvement in chromatin conformation during development of both asexual and sexual forms of the parasite (Fig. 4a).

Notably, modifications associated with gene activation cluster together and correlate clearly with the transcriptionally active schizonts during asexual development, including the novel modifications H3K4ac and H3K122ac. However, once the parasite is committed to gametocytogenesis, early stage I–III gametocytes are characterised by abundant repressive modifications including H4K20me3 and H3K36me2. For the first time, we also observe the repressive modification H3K27me3 in Plasmodia, particularly abundant in stage II gametocytes^58^. This modification has previously been thought to be absent in Plasmodia, but in those studies only asexual forms of the parasite were investigated^39, 51^. Additionally, the repressive modification H3K9me3, show a marked increase in abundance throughout gametocytogenesis, but particularly so in early gametocytes. When the parasites then enter the mature stages of gametocytogenesis, abundant activation marks such as H3K4me1 (regulatory element modification), H3K18ac, H3K27me1 and H3K36me1 arise, and limited repression modifications such as H3K79me3 (elongation modification) and H3K79me1 are present. These stage-specific trends were statistically confirmed, with H3K4me1 modifications significantly (*P* < 0.001, n = 3) abundant in schizonts and gametocytes compared to other life cycle stages; H3K9me1 (*P* < 0.01, n = 3) and me2 (*P* < 0.001, n = 3) were present at significantly increased abundance in asexual stages compared to gametocytes (Fig. 4b). Histone PTM-specific Western blotting was performed over the eight harvested life cycle stages for histone H3K9ac and H4ac (K5 + K8 + K12 + K16) to determine the distribution during life cycle development. H4 acetylation is significantly (*P* < 0.001, n = 3) more abundant in gametocyte stages than asexual stages, with the exception of H4K16ac, which is highly abundant in all stages, but peaking in transcriptionally active schizonts. This data correlates with the Western blot results, validating this peaking trend in schizonts and gametocytogenesis.

Taken together, we show confirmation of the presence and abundance of stage-specific histone PTMs across both asexual and sexual *P. falciparum* life cycle development by confident spectral histone PTM assignments. This study confirms that the histone PTM landscape in *P. falciparum* is highly dynamic with clear stage-specific developmental trends associated with either asexual or sexual development.

## Discussion

With this large-scale chromatin proteomics study, we quantitatively identified a set of PTMs on histones in all life cycle forms of *P. falciparum* parasites, during both asexual proliferation and sexual differentiation. We confirmed the presence of several previously described modifications but also identified a set of completely new modifications. Moreover, we showed that histone PTMs have dynamic profiles associated with life cycle progression in malaria parasites and that there is a clear distinction between modifications employed during asexual proliferation and sexual differentiation.

Our strategy was based on stringent quantification of histone PTMs, to enable the horizontal comparison between datasets and allow extensive high-resolution dynamic analyses of the histone PTM landscape. Using three independent biological replicates, quantitative MS profiling and strict evaluation of individual PTMs, inaccurate assignments were avoided and assisted in the quantification of isobaric histone peptides^53^. Through this sensitive and stringent profiling, we were able to prove that only a proportion (20%) of 232 recently identified histone PTMs in *Plasmodium* ^50^ was biologically associated with life cycle progression. However, we cannot exclude the possibility that other types and positions of histone PTMs may still exist in *Plasmodium* in addition to those described in this study.

The global histone PTM landscape was shown to be highly dynamic and adaptable across the entire life cycle of the parasite, for all 46 marks quantified. Such a dynamic nature was previously eluded, but limited to only eight histone PTMs described in the parasite during asexual proliferation (H3K4me3, H3K9ac, H3K14ac, H3K56ac, H4K8ac, H4K16ac, H4ac4 and H4K20me1)^21^. These modifications were shown to be involved in euchromatin formation and are directly linked to transcriptional regulation during the IDC^21^ in support of our study showing distinct stage-specific dynamics. However, the dynamics in the histone PTMs are not limited to these eight modifications but are a more global phenomenon. An additional 18 modifications were shown to increase during asexual development. Association of these marks with genome occupancy^21^ and transcript expression would reveal their involvement and importance to global transcriptional regulation in the parasite.

The dynamic distribution of histone PTMs over the parasite’s life cycle therefore suggests a distinct mode of transcriptional regulation in the parasite and reveals an interesting association with transcriptional activation/silencing during life cycle progression. A high level of acetylation and mono-methylation is observed in our dataset (61% of all quantified, validated modifications), particularly associated with the asexual stages. These marks correlate to the transcriptionally permissive, euchromatic state of chromatin in the parasite’s asexual IDC^39, 50, 64^ and confirms that *P. falciparum* histones contain more euchromatic than heterochromatic modifications^29, 30, 32, 34, 36, 40, 42, 45, 57, 59–63, 65^, a phenomenon observed in most eukaryotes. Known activation marks (H3K4me2, H3K4me3, H3K9ac, H3K14ac and H4ac) increase in abundance as the parasite progresses from trophozoites to schizonts. This can be associated with increased transcriptional activity of the parasite of a previously suggested 76% of the *P. falciparum* genome during the IDC^21^. Previous reports showed peak occupancy of H3K4me3 during trophozoite development that strongly correlates with transcriptional expression evenly distributed along the IDC^21^. Here we show increased abundance for H3K4me3 in schizont stages, previously associated with promoters of active metabolism and invasion related genes^25, 27^.

In contrast to the transcriptionally permissive state associated with high levels of histone acetylation and mono-methylation marks of asexual stages, an increased abundance of di- and tri-methylation marks is observed during gametocytogenesis. These methylations are typically associated with the core histones, particularly H3 and are generally indicative of heterochromatic nuclei in a transcriptionally repressive state^34, 42, 45, 57, 63^. Their presence in gametocytes (particularly early gametocyte development) could indicate an associated heterochromatic status in these parasites, which we are currently investigating. Histone PTMs that have previously been shown to be associated with transcriptional silencing were confirmed in our study to be more abundant in earlier asexual stages. H3K9me3, the well-known PTM marking heterochromatic islands in clonally variant gene families involved in immune evasion^21, 27, 60, 63^, had an increased abundance during gametocytogenesis, particularly during early gametocyte development. Likewise, the recently identified global repressive mark H3K36me2^51^ was also shown here to have a higher abundance in early stage gametocytes. Another repressive mark, H3K27me3, was abundantly present in early stage II gametocytes but was not previously detected in studies using only asexual forms of the parasite^50, 51^. Stage II gametocytes also showed peak abundance of H3K36me3, a known *var* gene silencing mark in *P. falciparum* parasites^31^. An active *var* gene is characterised by, amongst other PTMs, H3K4me2 that is shown here to be highly abundant in schizonts^36^. This might suggest an inactive *var* gene expression state in the early stages of gametocyte development, strongly correlating to recent work showing downregulated *var* genes found in stage II gametocytes^66^. Various known euchromatin-associated modifications (e.g. H3K9ac, H3K14ac and H4ac) that are involved in parasite growth, metabolism and host-parasite interactions^23, 38, 39, 44^, were shown here to be present in the sexual developmental stages as well. A number of modifications (H4K20me3, H3K27ac and H3K4me1) with increased abundance during early gametocyte development were also associated with genes in a prepared state for transcription, but not yet fully transcribed^51^. Taken together, early gametocyte development show histone PTMs indicative of a G_0_ cellular stage^6^, similar to ring stage asexual parasites.

Another interesting stage-specific observation was the abundance of euchromatic histone PTMs in the mature stages (stage IV/V) of gametocytogenesis. Schizogony is known to involve high levels of transcription, however, a decreasing transcriptional activity was previously indicated as gametocytes progress to maturity^67^. Additionally, translational repression has been linked to the regulation of gene expression in gametocytogenesis, where the ‘quiescent’ mature gametocytes are said to store translationally repressed mRNAs to be used for subsequent developmental changes upon activation i.e. the onset of gametogenesis^68, 69^. The presence of activation modifications like H3K27me1 and H4K36me1 might be associated with a poised instead of a repressed transcriptional state, where stage IV/V gametocytes contain activation PTMs but show a lack of transcription, awaiting activation in the mosquito midgut. This would correlate to the activity of these marks found in other model organisms^70, 71^. H4ac (K5, K8, K12 & K16), a set of activation marks that has also been linked to the poised state of *var* genes in combination with H3K9me3^51^, was found to be more abundant during gametocytogenesis compared to asexual proliferation, further inferring toward a poised transcriptional state.

In totality, the comprehensive PTM landscape included 15 novel histone PTMs in Plasmodia. The schizont-associated novel PTMs, H3K4ac and H3K122ac, have been described in mammalian cells and *Saccharomyces* to define genetic elements involved in active transcription^72, 73^, suggesting this association can be extrapolated to this transcriptionally active stage of Plasmodia. Transcriptional poised states of stage V gametocytes are again linked to H3K27me1 & H3K36me1^70, 71^. Novel repressive marks have a wide distribution over the life cycle, including H3K27me2&3 (stage II gametocytes), H3K56me3 (schizonts, stage II & IV gametocytes) and H3.3K9me3 (rings & trophozoites)^58, 70, 74–76^.

Although this data links the presence of individual histone PTMs to specific life cycle compartments, the occurrence of co-existing PTMs is of particular interest. In eukarya, histone PTMs have been hypothesised to form a ‘histone code’, implying that dynamic patterns of combinations of histone PTMs correlate with specific chromosomal states and binding of regulatory proteins^77, 78^, thereby coordinating epigenetic gene regulation and development^79^. We accurately quantified 30 co-existing histone PTMs over the parasite’s life cycle, including acetylation of histone H4 at four different lysine residues (K5, K8, K12 and K16) on a single peptide, resultant of simultaneous acetylation by the histone acetyltransferase PfMYST^32^. This co-occurrence of H4ac is particularly evident in gametocyte stages, and corresponds to the increased expression of PfMYST during gametocytogenesis (results not shown). The co-occurrence of H3K9me3 and H3K14ac also confirms previous findings that the newly characterised methyltransferase (PfSET7) methylates H3K9 with pre-existing H3K14ac^80^. The co-occupancy of genomic loci for H3K4me3 and H3K9ac^51^ is also justified by the presence of both PTMs during life cycle development. Various other combinations were also identified, including combinations of novel *P. falciparum* histone PTMs e.g. H3K18me1 & H3K23me1, H3K27me1-3 & known H3K36me1-3 and H3.3K9me1-3 & H3.3K14ac. The functional significance of these co-occurrences is currently under investigation.

Our study reveals unique plasticity and dynamics in the histone PTM landscape of *P. falciparum* parasites, which is associated with life cycle progression and differentiation. This first description of the extensive, quantitative histone PTM landscape of *P. falciparum* parasites and the wide array of histone PTMs found in the various life cycle stages supports the notion that the epigenome of the *P. falciparum* parasite is complex and adaptive during development, and might be the parasite’s Achilles heel^1, 28, 41, 44, 47^. Epigenetic regulatory machinery is deemed an essential target for chemotherapy, as cancer development has been widely linked to epigenetic regulation by histone PTMs, their regulating enzymes and histone-binding proteins^81, 82^. For *Plasmodium*, we need to reach a similar level of understanding concerning the epigenetic regulatory processes of parasite development on a molecular level, in order to enhance diagnosis and treatment strategies.

## Methods

### Parasite production and isolation

*P. falciparum* 3D7 (drug sensitive) and *P. falciparum* NF54-PfS16-GFP-Luc (kindly provided by the Fidock lab, Columbia University, USA)^83^ parasite cultures were maintained at 5% haematocrit in O^+^ human erythrocytes and RPMI-1640 cell culture medium supplemented with 24 mM sodium bicarbonate, 0.024 mg/ml gentamicin, 25 mM HEPES, 0.2% v/v glucose, 0.2 mM hypoxanthine and 0.5% w/v AlbuMAX II Lipid Rich Bovine Serum Albumin. The parasite cultures were kept at 37 °C with moderate shaking at 60 rpm and gassed with a mixture of 5% O_2_, 5% CO_2_ and 90% N_2_^84^. Synchronous asexual parasites (>90%, Pf3D7) were obtained using 10% w/v D-sorbitol^85^ and isolated as ring, trophozoite and schizont stages at 14, 32 and 42 hours post invasion (hpi), respectively. Gametocytes were induced from a >90% synchronised asexual *P. falciparum* NF54-PfS16-GFP-Luc culture (described previously^86^, maintained in culture medium lacking glucose) at 0.5% parasitaemia (6% haematocrit) by enforcing environmental stress to asexual parasites^87^. Gametocyte cultures were maintained for 12 days, treated with 50 mM *N*-acetyl-D-glucosamine and the stage I, II, III, IV and V gametocytes were isolated on day 3, 5, 7, 9 and 12 of gametocytogenesis, respectively. The asexual and sexual parasites were released from the RBCs using 0.06% w/v saponin in phosphate buffered saline (PBS), followed by several wash steps with PBS to eliminate the presence of any residual erythrocyte material.

### Histone isolation and chemical derivatisation

Histones were isolated using a modified protocol from Trelle *et al*.^39^. Briefly, nuclei was released from the pure and isolated *P. falciparum* parasites using a hypotonic buffer containing 10 mM Tris-HCl, pH 8.0, 3 mM MgCl_2_, 0.2% v/v Nonidet P40, 0.25 M sucrose in the presence of a protease inhibitor cocktail. The mixture was centrifuged at 500 *g* and 4 °C for 10 min and this hypotonic buffer wash step was repeated twice. Subsequently, the chromatin pellet was homogenised in the hypotonic buffer lacking Nonidet P40, to which 10 mM Tris-HCl, pH 8.0, 0.8 M NaCl, 1 mM EDTA (including protease inhibitor cocktail) was added, followed by a 10 min incubation on ice. Histones were acid-extracted from the chromatin with 0.25 M HCl and rotation at 4 °C for 1 h. The histone-containing supernatant was mixed with an equal volume of 20% v/v trichloroacetic acid (TCA), incubated on ice for 15 min and pelleted. The histone-enriched pellet was washed with acetone, air-dried and reconstituted using dddH_2_O. All samples were dried using a SpeedVac concentrator (SC100, Savant) and reconstituted with 100 mM ammonium bicarbonate (pH 7–9). Histones were prepared for MS analysis via propionic anhydride chemical derivatisation and in-solution trypsin digestion as previously described^88^. Each of the eight stages was analysed in three independent biological experiments with three technical triplicates each. For the initial semi-quantitative study, the experimental procedures are outlined in Supplementary Methods.

### Quantitative nanoLC-MS/MS-based histone PTM identification

Samples were analysed by using a nanoLC-MS/MS setup. NanoLC was configured with a 75 µm ID × 17 cm Reprosil-Pur C18-AQ (3 µm; Dr. Maisch GmbH, Germany) nano-column using an EASY-nLC nanoHPLC (Thermo Scientific, San Jose, CA, USA). The HPLC gradient was 0–35% solvent B (A = 0.1% formic acid; B = 95% acetonitrile, 0.1% formic acid) over 40 min and from 34–100% solvent B in 30 min at a flow-rate of 300 nL/min. The LC was coupled with an LTQ-Orbitrap Velos Pro mass spectrometer (Thermo Scientific, San Jose, CA, USA). Full scan MS spectrum (*m/z* 290–1400) was performed in the Orbitrap with a resolution of 60,000 (at 400 m/z) with an AGC target of 1 × 10^6^. The 10 most intense ions at each cycle were selected for MS/MS performed with collision induce dissociation (CID) with normalised collision energy of 35, an automatic gain control (AGC) target of 10^4^ and a maximum injection time of 100 ms. MS/MS data were collected in centroid mode in the ion trap. Precursor ion charge state screening was enabled and all unassigned charge states, as well as singly charged species, were rejected. The dynamic exclusion list was restricted to a maximum of 500 entries with a maximum retention period of 30 s. In selected time windows we included targeted scan events for isobarically modified peptides, in order to profile their fragment ions and discriminate their abundance, which cannot be performed with just the full MS chromatogram.

### Data analysis

Modified histone peptides were identified by processing and searching MS/MS spectra and searching DDA runs with Proteome Discoverer (v1.4, Thermo Scientific, Bremen, Germany). Mascot was used as the database searching engine, using PlasmoDB v24 (03/2015). Search parameters were as follows: MS tolerance: 10 ppm; MS/MS tolerance: 0.5 Da; enzyme: trypsin; static modifications: propionyl N-termini; dynamic modifications: methyl (+propionyl), dimethyl, trimethyl, acetyl, and propionyl (K). Peptides were filtered for high confidence (FDR < 1%) using Fixed Value validator. Quantification was performed using EpiProfile^53^, which performs extracted ion chromatography of selected peptides. Once the peak area was extracted, the relative abundance of a given PTM was calculated by dividing its intensity by the sum of all modified and unmodified peptides sharing the same sequence. For trend analyses data were z-scored. Trend analysis and statistical regulations of PTMs across the developmental stages was assessed using multiple tests: (i) the Mann Kendall test was used to verify which marks have a significant monotonic trend (constantly increasing or decreasing); (ii) k-means clustering was used to group marks that have very similar trends between conditions; (iii) Co-expression correlation was used to compare every single modification with one another and by sorting such data, histone modifications that had the most similar and most different regulation, were verified; (iv) The Kolmogorov Smirnov test was used to verify whether a trend was random or with clear slopes in specific time points.

Correlation of the histone yield with histone expression levels in *P. falciparum* were performed using full transcriptome data for asexual parasites (48 h IDC, H. Painter) and gametocytes (R. van Biljon, submitted elsewhere); and proteomics data (N. Coetzee, manuscript in preparation).

### Western Blot validations

Isolated histones were separated on a 4–20% SDS-PAGE gel and transferred to a PVDF membrane (iBlot^®^ 2 PVDF Mini stacks, Invitrogen) using the iBlot^®^ 2 dry blotting system on program P0 for 7 min. Membranes were blocked in TBS-T (50 mM Tris-HCl, pH 7.5, 150 mM NaCl, 0.1% Tween-20) containing 5% milk powder and probed for H3K9ac (Abcam ab61231; 1:5000) or H4ac (K5 + K8 + K12 + K16 acetyl; Abcam ab10807; 1:5000) primary antibodies diluted in TBS-T. This was followed by anti-rabbit HRP (Abcam ab97095; 1:5000) secondary antibody and visualisation using Pierce SuperSignal^®^ West Pico chemiluminescent substrate.

## Electronic supplementary material

Supplementary information and figures

Supplementary File S1

Supplementary File S2

Supplementary File S3

Supplementary File S4
