# Supplementary material for: Quantitative chromatin proteomics reveals a dynamic histone post-translational modification landscape that defines asexual and sexual Plasmodium falciparum parasites
Source: Sci Rep. 2017 Apr 4;7:607. doi: 10.1038/s41598-017-00687-7 (PMC5428830; doi:10.1038/s41598-017-00687-7)
Supplement: Supplementary file 1 — Supplementary information and figures [file 41598_2017_687_MOESM1_ESM.pdf]

Quantitative chromatin proteomics reveals a dynamic histone post-translational modification landscape that defines asexual and sexual *Plasmodium falciparum* parasites

Nanika Coetzee[1], Simone Sidoli[2], Riëtte van Biljon[1], Heather Painter[3], Manuel Llinás[3], Benjamin A. Garcia[2] and Lyn-Marie Birkholtz[1]*

[1] Department of Biochemistry, Centre for Sustainable Malaria Control, University of Pretoria, Private bag x20, Hatfield, Pretoria, South Africa

[2] Epigenetics Program, Department of Biochemistry and Biophysics, Perelman School of Medicine, University of Pennsylvania, Philadelphia, Pennsylvania, United States of America

[3] Department of Biochemistry & Molecular Biology and Centre for Malaria Research, Pennsylvania State University, University Park, Pennsylvania, United States of America

* Corresponding author:

E-mail: lbirkholtz@up.ac.za (LB)

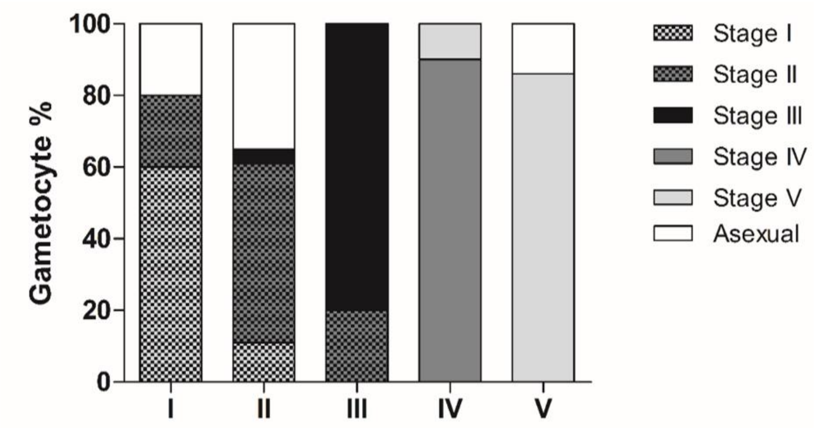

Supplementary Figure S1. Gametocyte-stage composition. From the isolated parasites, the stage I, II, III, IV and V gametocytes were shown to have 60%, 50%, 80%, 90% and 86% synchronicity, respectively. The contaminating asexual stages were seen to be mostly earlier asexual stages, therefore contributing minimal to the histone prevalence.

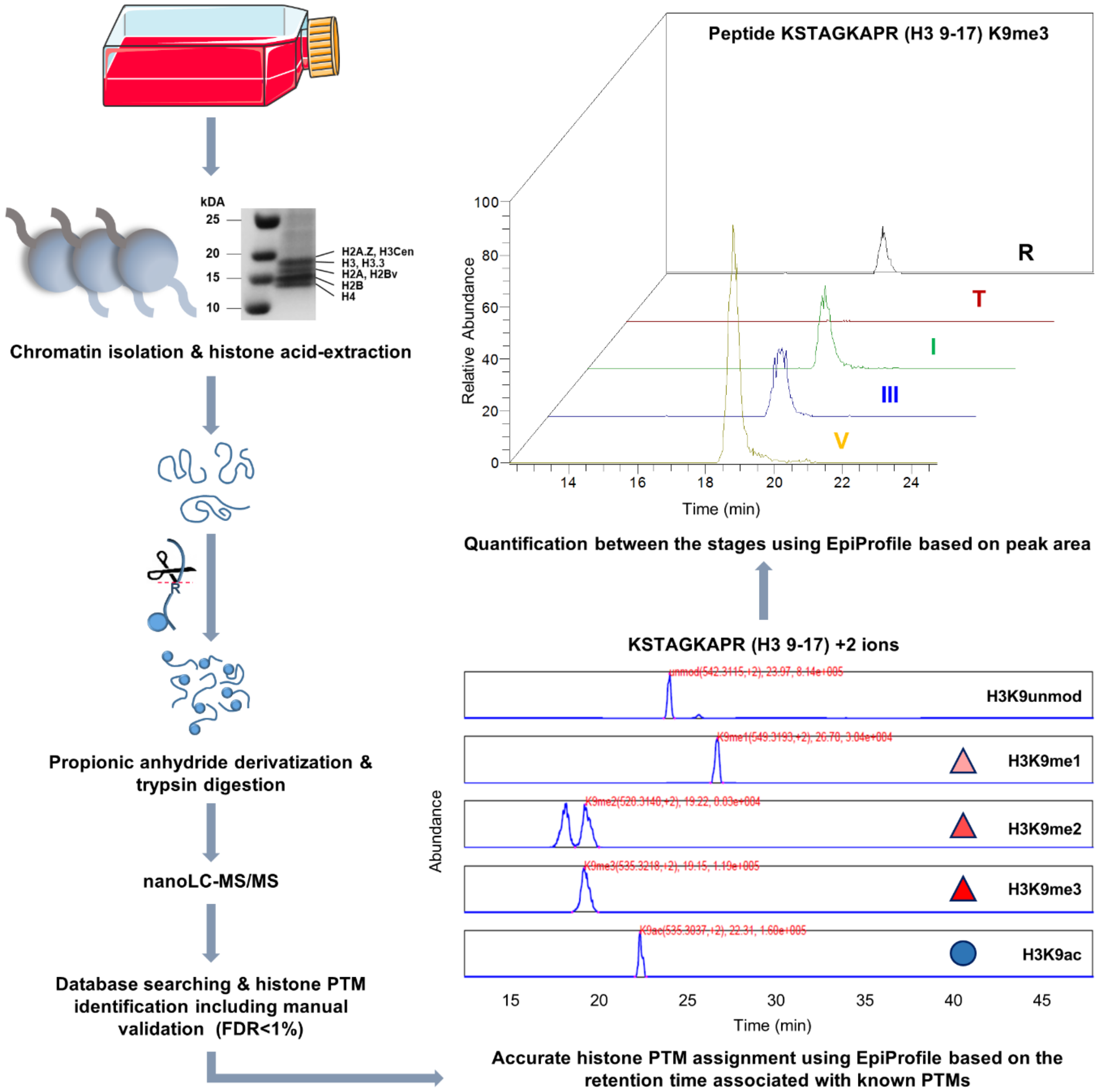

Supplementary Figure S2. Chromatin proteomics approach for histone PTM quantification. The core and variant histones from each of the eight representative life cycle stages were acid-extracted, propionylated and trypsin digested. NanoLC-MS/MS was performed, followed by database searching and histone PTM identification including manual validation (FDR<1%). MS1 spectra of the *P. falciparum* histone H3 peptide KSTAGKAPR (aa 9-17), illustrating the discrimination between varying histone PTMs of H3K9 based on the retention time associated with known PTMs. After accurate PTM assignments, relative quantification using EpiProfile was performed, enabling accurate quantification of histone PTMs based on peak area and determination of co-existing PTMs on longer peptides. The abundance profiles during life cycle development were derived in this manner. The culture flask image was adapted from Servier Medical Art (URL link to the license: https://creativecommons.org/licenses/by/3.0/) and changes were made in terms of colour and size.

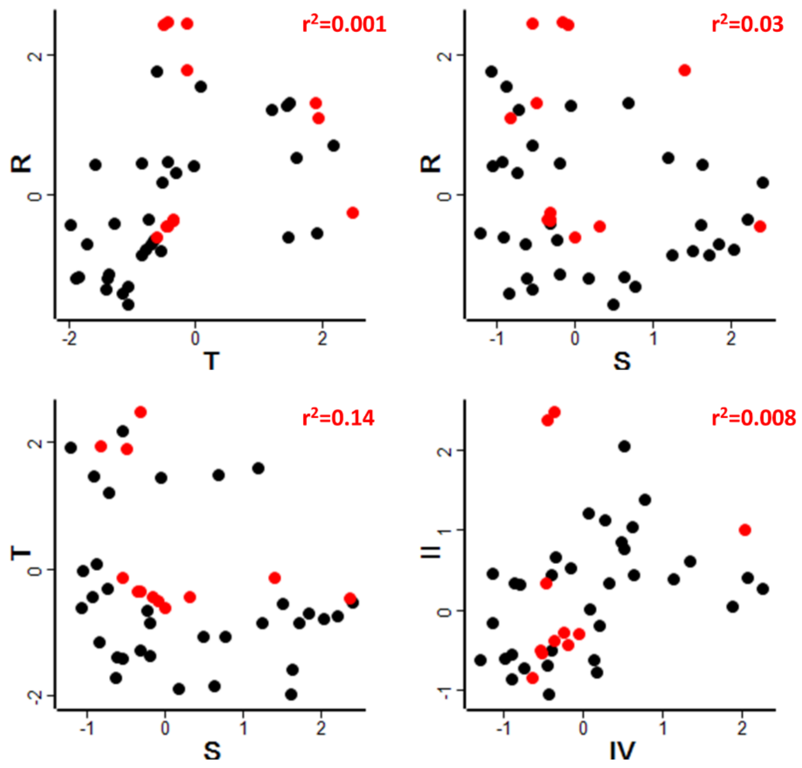

Supplementary Figure S3. Anti-correlation of conserved histone PTMs compared to stage I, III & V gametocytes. Correlation of a set of conserved residues (H3K4ac, H3K9me1, H3.3K9me2, H3K18me1, H3K23me1, H3K27me2 & me3, H3K36me2 & me3, H3K56me1 & me2, H3K79me3 and H3.3K79me3) between rings, trophozoites, schizonts and stage II & IV gametocytes are shown (r^2 values are indicated as 0.001, 0.03, 0.14 and 0.008).

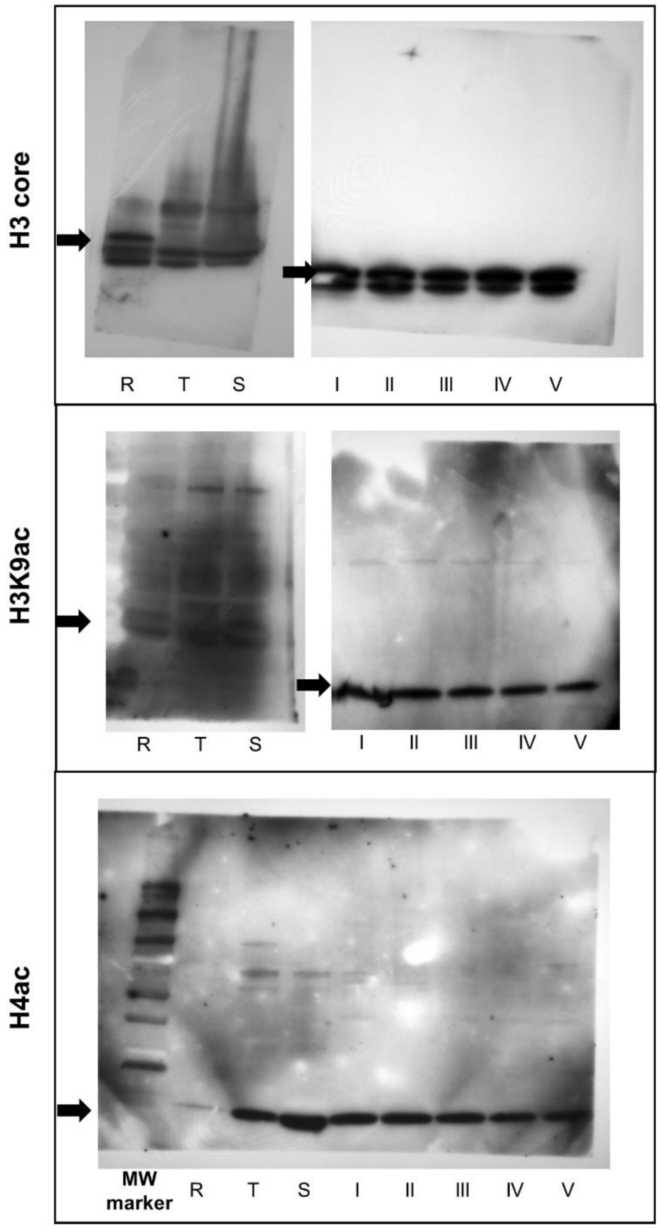

Supplementary Figure S4. Full-length western blots for H3Core, H3K9ac and H4ac. The Precision Plus Protein^{TM} Dual Color Standard was used throughout the experiments (10-250 kDa) as molecular weight marker. Exposure times varied due to differences in antibody sensitivity. Developing and fixing times were constant at 30 s each. All samples were derived from the same experiments and gels/blots were processed in parallel.

Supplementary Methods

Parasite production and isolation

P. falciparum NF54 (drug sensitive) parasite cultures were maintained at 5% hematocrit in O^+ human erythrocytes and RPMI-1640 cell culture medium supplemented with 24 mM sodium bicarbonate, 0.024 mg/ml gentamicin, 25 mM HEPES, 0.2% v/v glucose, 0.2 mM hypoxanthine and 0.5% w/v AlbuMAX II Lipid Rich Bovine Serum Albumin. The parasite cultures were kept at 37ºC with moderate shaking at 60 rpm and gassed with a mixture of 5% O_2, 5% CO_2 and 90% N_2. The asexual parasites were tightly synchronized with 10% w/v D-sorbitol and isolated at various time points. *P. falciparum* gametocytes were induced from a >90% synchronized asexual culture (maintained in culture medium lacking glucose) at 0.5% parasitemia (6% hematocrit) by enforcing environmental stress to asexual parasites as described previously. Gametocyte cultures were maintained for 14 days, where the early (Day 8, stage I-III) and late gametocytes (Day 14, stage IV-V) were treated with 50 mM *N*-acetyl-D-glucosamine on days 5-7 and days 9-11, respectively. The early and late gametocytes were isolated and enriched with magnetic separation (MACS® LS columns) to 90%. The asexual and sexual parasites were released from the RBCs using 0.06% w/v saponin in phosphate buffered saline (PBS), followed by several wash steps with PBS.

Histone isolation and separation

Histones were acid-extracted using a modified protocol from Trelle *et al.* (2009). All samples were kept on ice throughout the isolation protocol. The isolated *P. falciparum* parasite nuclei were released using a hypotonic buffer containing 10 mM Tris-HCl, pH 8.0, 3 mM $MgCl_2$, 0.2% v/v Nonidet P40, 0.25 M sucrose in the presence of a protease inhibitor cocktail. The mixture was centrifuged at 500*g* at 4ºC for 10 min and this hypotonic buffer wash step was repeated twice. Subsequently, the pellet was homogenized in the hypotonic buffer lacking NP-40 to obtain pelleted nuclei. To this, 10 mM Tris-HCl, pH 8.0, 0.8 M NaCl, 1 mM EDTA (including protease inhibitor cocktail) was added, followed by a 10 min ice incubation. Histones were acid extracted from the nuclei with 0.25 M HCl and rotation at 4ºC for 1 h. The histone-containing supernatant was mixed with an equal volume of 20% v/v trichloroacetic acid, incubated

on ice for 15 min and pelleted. The histone-enriched pellet was washed with acetone, air-dried and resuspended in SDS sample buffer (62.5 mM Tris-HCl pH 6.8, 8.7% (v/v) glycerol, 2% (w/v) SDS, 0.2% (w/v) bromophenol blue and 0.7% (v/v) 2- β-mercaptoethanol). The samples were run on a 4-20% separating SDS-PAGE and stained using coomassie blue R-250. Excised protein bands were destained overnight (50% (v/v) acetonitrile, 50 mM ammonium bicarbonate), after which in-gel digestion was performed overnight with 10 ng/µl trypsin at 37ºC. Peptides were extracted from the gel matrix with a 30 min incubation in 70% acetonitrile, 0.1% trifluoroacetic acid and another 30 min incubation in 100% acetonitrile, 0.1% trifluoroacetic acid. The peptides were dried and reconstituted in 5% (v/v) formic acid and cleaned using Stage Tips. The peptides were dried again and dissolved in 5% acetonitrile and 0.1% formic acid, after which 10 µl injections were made for nano-LC chromatography.

Qualitative LTQ-MS/MS histone PTM identification

All experiments were performed on a Thermo Scientific EASY-nLC II connected to a LTQ Orbitrap Velos mass spectrometer (Thermo Scientific, Bremen, Germany) equipped with a nano-electrospray source. For liquid chromatography, separation was performed on an EASY-Column (2 cm, ID 100µm, 5 µm, C18) pre-column followed by an XBridge BEH130 NanoEase column (15 cm, ID 75 µm, 3.5 µm, C18) with a flow rate of 300 nl/min. The gradient used was from 5-17% B in 5 min, 17-25% B in 90 min, 25-60% B in 10 min, 60-80% B in 5 min and kept at 80% B for 10 min. Solvent A was 100% water in 0.1 % formic acid, and solvent B was 90 % acetonitrile in 0.1% formic id. The mass spectrometer was operated in data-dependent mode to automatically switch between Orbitrap-MS and LTQ-MS/MS acquisition. Data were acquired using the Xcalibur software package. The precursor ion scan MS spectra (*m/z* 400-2000) were acquired in the Orbitrap with resolution R = 60000 with the number of cumulated ions being 1x106. The 20 most intense ions were isolated and fragmented in linear ion trap (number of cumulated ions 1.5x104) using collision-induced dissociation. The lock mass option (polydimethylcyclosiloxane; *m/z* 445.120025) enabled curate mass measurement in both the MS and MS/MS modes. In data-dependent LC-MS/MS experiments, dynamic exclusion was used with 60 s exclusion duration. Mass spectrometry conditions were 1.8 kV, capillary temperature of 250°C, with no sheath and auxiliary gas flow. The ion selection threshold was 500 counts for MS/MS. An activation Q-value of 0.25 and activation time of 10 ms were also applied for MS/MS.

Thermo Proteome Discoverer 1.3 was used to identify proteins via automated database searching of all tandem mass spectra against the PlasmoDB 7.2 database, using SEQUEST. Carbamidomethyl cysteine was set as a fixed modification, with oxidized methionine, N-terminal acetylation, lysine acetylation, serine and threonine phosphorylation, lysine and arginine mono-, di- and trimethylation as dynamic modifications. The precursor mass tolerance and fragment mass tolerance was set to 20 ppm and 0.8 Da, respectively, with six missed tryptic cleavages allowed. Proteins were considered positively identified when they were identified with at least 1 tryptic peptide per protein, a Mascot score threshold of 20 and a SEQUEST X-score threshold of 1.5. Percolator was used for peptide validation with a maximum ΔCn of 0.5, and decoy database searches with a false discovery rate of 0.02 and 0.05, with validation based on the q-value. All the identified PTMs were manually validated against the fragment match spectrum of every identified peptide containing the PTMs of interest to establish the significance of every modification.

Supplementary files

Supplementary File S1 - Quantitative list of peptides.

Supplementary File S2 - All identified PTMs.

Supplementary File S3 - Quantitative histone PTM landscape.

Supplementary File S4 - Co-existing PTMs.
